# Matrix Metalloproteinase 7 Expression and Apical Epithelial Defects in *Atp8b1* Mutant Mouse Model of Pulmonary Fibrosis

**DOI:** 10.3390/biom12020283

**Published:** 2022-02-09

**Authors:** Emma Westermann-Clark, Ramani Soundararajan, Jutaro Fukumoto, Sahebgowda Sidramagowda Patil, Timothy M. Stearns, Smita Saji, Alexander Czachor, Helena Hernandez-Cuervo, Mason Breitzig, Sudarshan Krishnamurthy, Richard F. Lockey, Narasaiah Kolliputi

**Affiliations:** 1 Laboratory of Dr. Narasaiah Kolliputi, Department of Internal Medicine, Division of Allergy/Immunology, College of Medicine, University of South Florida Morsani, Tampa, FL 33612, USA; ewester1@usf.edu (E.W.-C.); ramanis@usf.edu (R.S.); fukumoto_kokyu@yahoo.co.jp (J.F.); sahebgowda@mail.usf.edu (S.S.P.); smitasaji74@gmail.com (S.S.); czachor@mail.usf.edu (A.C.); mariahelena@usf.edu (H.H.-C.); mbreitzig@usf.edu (M.B.); sukrishn@wakehealth.edu (S.K.); rlockey@usf.edu (R.F.L.); 2 Departments of Medicine and Pediatrics, Division of Allergy/Immunology, College of Medicine, University of South Florida, Tampa, FL 33612, USA; 3 MDI Biological Laboratory, Salisbury Cove, ME 04672, USA; tim.stearns@jax.org; 4 Wake Forest School of Medicine, Bowman Gray Center for Medical Education, Winston-Salem, NC 27101, USA

**Keywords:** idiopathic pulmonary fibrosis, *Atp8b1* mutant, MMP7, apical, ciliogenesis

## Abstract

Abnormalities in airway epithelia and lung parenchyma are found in *Atp8b1* mutant mice, which develop pulmonary fibrosis after hyperoxic insult. Microarray and ingenuity pathway analysis (IPA) show numerous transcripts involved in ciliogenesis are downregulated in 14-month (14 M) -old *Atp8b1* mouse lung compared with wild-type C57BL/6. Lung epithelium of *Atp8b1* mice demonstrate apical abnormalities of ciliated and club cells in the bronchial epithelium on transmission electron microscopy (TEM). Matrix metalloproteinase 7 (MMP7) regulates of ciliogenesis and is a biomarker for idiopathic pulmonary fibrosis (IPF) in humans. *Mmp7* transcript and protein expression are significantly upregulated in 14 M *Atp8b1* mutant mouse lung. MMP7 expression is also increased in bronchoalveolar lavage fluid (BAL). Immunohistochemistry is localized MMP7 to bronchial epithelial cells in the *Atp8b1* mutant. In conclusion, MMP7 is upregulated in the aged *Atp8b1* mouse model, which displays abnormal ciliated cell and club cell morphology. This mouse model can facilitate the exploration of the role of MMP7 in epithelial integrity and ciliogenesis in IPF. The *Atp8b1* mutant mouse is proposed as a model for IPF.

## 1. Introduction

Idiopathic pulmonary fibrosis (IPF) is a poorly understood disease that historically led to death within 3–5 years of diagnosis [1], although antifibrotic medications, such as nintedanib, can significantly extend life expectancy [2,3]. Specific biomarkers, especially matrix metalloproteinase 7 (MMP7), are implicated in the pathogenesis of IPF, but the mechanisms by which MMP7 may contribute to fibrosis remain unclear.

MMP7 is a serum biomarker for preclinical disease in IPF in humans [4]. Serum MMP7 distinguishes IPF from other types of interstitial lung disease with a sensitivity of 71.7% (range, 71–72.3%), specificity of 64.4% (63–66.3%), and diagnostic odds ratio of 4.7 (4.2–5.1) [5]. Despite these favorable test characteristics, guidelines published in 2018 from the American Thoracic Society do not support measuring serum MMP7 to diagnose IPF because of concern about false negatives, which could limit patient access to life-prolonging therapies [5]. Plasma MMP7 concentrations are significantly higher in IPF patients than in healthy controls, (14.40 ± 6.55 ng/mL vs 6.03 ± 2.51 ng/mL, *p* < 0.001) and a threshold of >12.1 ng/mL predicts mortality [6,7]. Multiple investigators conclude that MMP7 has diagnostic and prognostic value, sometimes in combination with other biomarkers [8,9,10,11,12,13,14]. MMP7 also may correlate with disease severity, as measured by diffusion capacity of the lungs for carbon monoxide % (DLCO%) and forced vital capacity % (FVC%) [4,15]. MMP7 knockout mice are protected from bleomycin-induced fibrosis [16]. MMP7 expression is increased in migrating epithelial cells in both human and mouse trachea after injury [17]. These studies support a role for MMP7 in pulmonary fibrosis.

Much of the animal research on IPF has been conducted using the bleomycin injury mouse model [18,19,20]. Bleomycin causes extensive inflammation and fibrosis in the mouse lung. While the bleomycin injury model is a widely used pre-clinical model for IPF, the reversible nature of bleomycin-induced fibrosis limits applicability in humans (Tashiro 2017) [21]. Therapeutic interventions tend to be administered shortly after bleomyin injury, which may bias it toward therapeutic interventions that reduce inflammation rather than reverse fibrosis.

The *Atp8b1* mouse model is an alternate model of IPF. The *Atp8b1* mouse displays morphologic changes after hyperoxic insult (48 h of hyperoxia) including aberrant proliferation of club cells at the bronchiolar epithelium [22]. During the recovery phase, under normoxia, a patchy distribution of interstitial fibrosis occurs, including cystic lesions with thickened interstitium and patchy parenchymal fibrosis [22].

*Atp8b1* is a P4-type ATPase or “flippase” that is thought to transport membrane phospholipids, including phosphatidylserine [23] and phosphatidylcholine [24]. Humans with *Atp8b1* defects have abnormalities in the apical membranes of several cell types including hepatocytes [25] and stereocilia of the ear [26]. The human clinical phenotype includes intrahepatic cholestasis leading to early cirrhosis, pruritis, growth failure, hearing defects, and recurrent pneumonia [27].

The reason for pulmonary fibrosis in the *Atp8b1* mouse is not clear. Given the emerging links between MMP7 and pulmonary fibrosis in humans, we investigate MMP7 levels in the *Atp8b1* mouse lung. Noting that MMP7^−^/^−^ mice are protected from bleomycin-induced fibrosis [16] and that their airway epithelia are carpeted with cilia after injury [28] we focus on the roles of MMP7 and ciliogenesis in the *Atp8b1* mouse model.

## 2. Materials and Methods

### 2.1. Animals

Dr. Laura Bull at the University of California at San Francisco generously provided the *Atp8b1^G308V/G308V^* mutant mouse on a C57Bl/6 background. Control C57BL/6 mice were obtained from Harlan laboratories (Indianapolis, IN, USA) (RRID:MGI:5656552). Animals were maintained at the University of South Florida in a specific-pathogen-free facility, as described previously [29]. The University of South Florida Institutional Animal Care and Use Committee (IACUC) approved animal protocols (Animal Welfare Assurance Number: A4100-01) according to “Guide for the Care and Use of Experimental Animals” from the National Institute of Health (NIH) (Revised 2011).

### 2.2. Collection of Mouse Lungs

Mice were anesthetized and underwent thoracotomy as described in [30]. Following lung removal, samples were fixed in formalin or flash frozen as described here [22,30].

### 2.3. Total RNA Extraction

Total RNA from C57BL/6 and *Atp8b1* mutant lungs was extracted using Trizol™ (Ambion^®^, ThermoFisherScientific, Carlsbad, CA, USA) and RNeasy kit (Qiagen, Hilden, Germany) as described in Soundararajan et al., 2016 [29].

### 2.4. Transcriptome Analysis of Mouse Lung

Transcriptome analysis was performed as described in [29]. The H. Lee Moffitt Cancer Center conducted the microarray analysis as described previously [29].

### 2.5. Differential Gene Expression

Affymetrix microarray analysis used to identify differentially expressed genes as previously described [29]. Differentially expressed genes were further analyzed using Ingenuity Pathway Analysis (IPA) [31], (Ingenuity Systems, Qiagen, Redwood City, CA, USA) as described previously [29].

### 2.6. Ingenuity Pathway Analysis

Ingenuity Pathway Analysis software [31] (IPA; Ingenuity Systems, Version 2020–2021, Qiagen.) was used to identify gene networks affected in C57BL/6 and *Atp8b1* mutant mice as described previously [29].

### 2.7. Quantitative Real-Time PCR (qPCR)

The iScript™ cDNA synthesis kit (Biorad Laboratories, Hercules, CA, USA) was used for reverse transcription. Mouse qPrimerDepot [32] was used for primer sequence (MMP7 forward primer: GCATTTCCTTGAGGTTGTCC; MMP7 reverse primer: CACATCAGTGGGAACAGGC;). Quantitative real time PCR was performed and analyzed as previously described [29].

### 2.8. Western Blot Analysis

Western blot analysis was performed on lung homogenates as described previously [22]. MMP7 antibody (a gift from Dr. Conor Lynch; antibody created in Dr. Lynn Matrisian’s laboratory) was used for overnight incubation at 4 °C [33]. Horseradish peroxidase-conjugated β-actin antibody (Sigma Aldrich, St Louis, MO, USA) was used as a loading control.

### 2.9. Immunohistochemistry

Single-color immunohistochemical (IHC) staining was performed on paraffin embedded *Atp8b1* and C57Bl/6 mouse lung tissue sections. described previously [22]. Sections were incubated with rabbit MMP7 antibody (Abcam 85144, Cambridge, MA, USA) at 4 °C overnight, with secondary antibody and detection as described previously [22].

### 2.10. Electron Microscopy

14 M *Atp8b1* (*n* = 3) and WT (*n* = 3) mouse lung samples, 1 mm in diameter, were fixed in 2% glutaraldehyde in sodium cacodylate. The samples were fixed overnight and then rinsed in cacodylate buffer. Samples were incubated for one hour in 1% osmium in 0.1M cacodylate buffer (pH 7.5). Ethanol gradients and acetone were used for the washing and sequential dehydration steps. Embed-812 (Electron Microscopy Sciences, Hatfield, PA, USA) was used as embedding resin, and an ultramicrotome (UCT; Leica, Wetzlar, Germany) was used to obtain thin sections ranging from 90–100 nm. A transmission electron microscope (JEM 1400; JEOL, Tokyo, Japan) with digital camera was used to examine samples at 80 kV (Gatan, Inc., Pleasanton, CA, USA).

### 2.11. Statistical Analysis

For RT-PCR, student’s *t*-test was used to calculate statistical significance between the two groups in Microsoft Excel. *p* values < 0.05 were considered to be statistically significant.

## 3. Results

### 3.1. Microarray Analysis Shows Decreased Expression of Ciliogenesis Genes

Microarray analysis revealed the decreased expression of genes that are involved in ciliogenesis. Some of the transcripts decreased in *Atp8b1* mutant mice at 14 M versus the 14 M WT controls included *Foxj1, Dnaaf1, Drc1, Dnah7b, Dnah7c, Dnah2, Dnah6, Efhc1, Dnali1* (Table 1). Differentially expressed cilia-related genes (*q* < 0.1, false discovery rate corrected *Mann–Whitney U* test *p*-value and median difference > 1.0) are listed in Table 1.

### 3.2. Ingenuity Pathway Analysis

**Figure 1:**
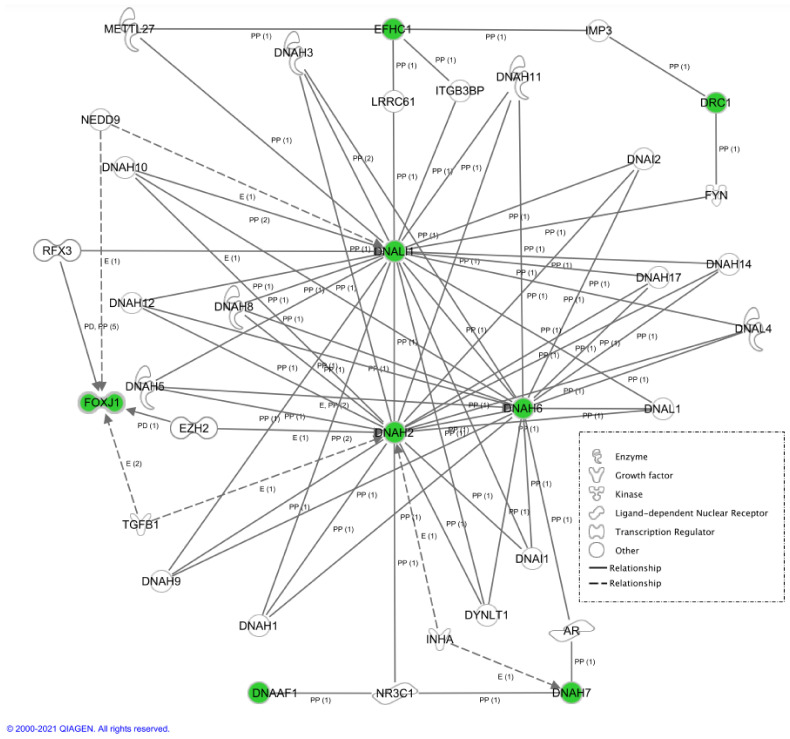
Network of genes involved in ciliogenesis, identified by Ingenuity Pathway Analysis (Ingenuity Systems, 2020–2021, Qiagen). In each network, the genes are represented as nodes. The biological relationship between two nodes is represented as an edge (line). Node color indicates up- (red) or down- (green) -regulation with respect to the datasets. The shape of each node represents the functional class of the gene product.

Functional analysis using Ingenuity Pathway Analysis (IPA) showed enrichment of transcripts involved in primary ciliary dyskinesia, the movement of cilia, the formation of cilia and the morphogenesis of cilia (Table 2, Figure 1).

### 3.3. Atp8b1 Mutant Mice Exhibit Abnormal Ciliogenesis

**Figure 2:**
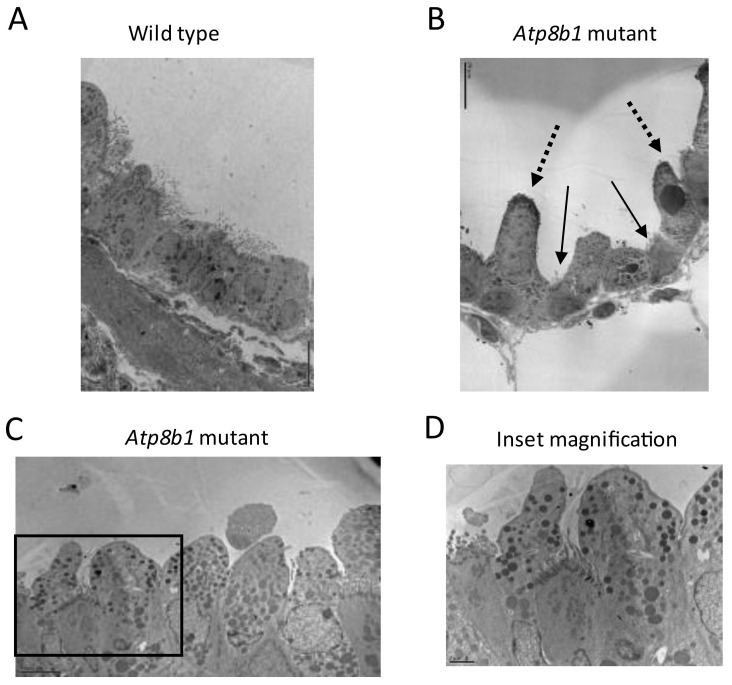
Representative electron microscopy images of bronchial epithelium (14 M old *Atp8b1* vs WT mouse lung). (**A**) TEM of WT lung shows normal bronchial epithelium, with ciliated cells interspersed with club cells. Ciliated and club cells in WT bronchial epithelium are approximately the same height, and club cells have a smooth border. (**B**) Bronchial epithelium of *Atp8b1* mouse lung: solid arrows indicate truncated ciliated cells and dotted arrows indicate protruding club cells. Magnification of Panel A is 2000×; magnification of Panel B is 2500×. Scale bars 10 μM. (**C**) Electron microscopic image of *Atp8b1* bronchial epithelium with ciliated cell submerged between club cells. Magnification is 4000×; scale bar 5 μM. (**D**) Inset from panel c.; magnification is 8000× with scale bar 2 μM.

The bronchial epithelia of *Atp8b1* and WT mice were examined using transmission electron microscopy (TEM). Ciliated cells lining the bronchial epithelium appeared to be truncated or submerged between abnormal-appearing club cells in *Atp8b1* mice (Figure 2A–D) at 14 M. The apical membrane of club cells also appeared abnormal in mutant mice; the normal club cell has a bulbous appearance with a smooth continuous border, whereas *Atp8b1* mutant mouse club cells have more irregular apical membranes.

### 3.4. Mmp7 mRNA and MMP7 Protein Significantly Increased in 14 M-Old Atp8b1 Mice

The change in ciliated cell morphology and apical changes in club cells observed on TEM prompted exploration of the role of ciliogenesis in pulmonary fibrosis, with a specific focus on MMP7. Transcription of MMP7 was evaluated in WT and *Atp8b1* mice using qPCR. MMP7 gene transcription is upregulated in *Atp8b1* mice versus WT mice, with a fold change of 4.30 (range 1.84–7.9, standard deviation 2.31) by 2^−ΔΔCT^ method (Figure 2A). Student’s *t*-test confirmed a statistically significant increase in the transcription of MMP7 in mutant versus WT mice; *p*-value = 0.018.

**Figure 3:**
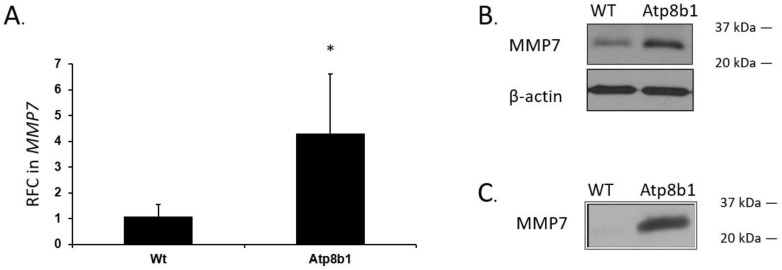
MMP7 is increased at both the mRNA and protein level in whole lung lysate of the *Atp8b1* mutant mouse relative to the WT control at 14 M, *n* = 6. (**A**) Quantitative real-time PCR (qPCR) of whole lung lysate and (**B**) Western blot demonstrate increased MMP7 at the mRNA and protein levels, respectively. (**C**) Western blot of bronchoalveolar lavage from 14 M mice also showed increased MMP7 in *Atp8b1* mice relative to WT mice. * *p* < 0.05. Abbreviations: WT: wild-type, RFC: relative fold change.

Western blot of lung lysate from WT and *Atp8b1* mice was performed to assess MMP7 expression at the protein level. MMP7 protein expression is elevated in the *Atp8b1* mouse compared to WT (Figure 3B). Western blot also shows that MMP7 is upregulated in the bronchoalveolar lavage fluid (BALF) of 14 M *Atp8b1* mice compared with WT mice (Figure 3C).

### 3.5. MMP7 Is Expressed in Bronchioles of Atp8b1 Mutant Mice

**Figure 4:**
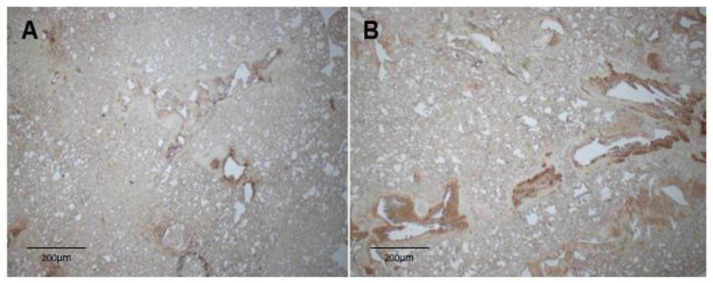
MMP7 expression in bronchial epithelium assessed by single-color immunohistochemistry using rabbit MMP7 antibody (Abcam 85144, Cambridge, MA, USA). (**A**) WT mouse lung with minimal MMP7 expression lining bronchioles. (**B**) *Atp8b1* mouse lung showing increased MMP7 expression lining bronchioles and scattered throughout alveoli.

Immunohistochemistry (IHC) was performed to localize the expression of MMP7 in lung tissue of WT and *Atp8b1* mice (Figure 4A,B). Preliminary immunohistochemistry shows that MMP7 is upregulated in *Atp8b1* mutant mice as compared with WT mice and localizes it to the bronchioles (Figure 4A,B). Taken together, mRNA transcript, protein expression, and IHC data show that MMP7 is upregulated in the airway epithelium of the *Atp8b1* mouse compared with WT mice.

## 4. Discussion

Abnormal morphology of ciliated cells and club cells in the *Atp8b1* mutant mouse and microarray data indicating decreased expression of ciliogenesis genes support a role for ciliogenesis in lung fibrosis in this mouse model. Ingenuity pathway analysis pointed to gene networks involved in primary ciliary dyskinesia, the movement of cilia, the formation of cilia and the morphogenesis of cilia. As MMP7 regulates ciliogenesis [28], we explored MMP7 expression in the *Atp8b1* mutant mouse. Human data support a role for MMP7 in IPF: the largest human IPF transcriptome analysis to date highlights *MMP7*, *MUC5b* and cilia genes as the most upregulated genes in IPF [34]. Serum MMP7 correlates with disease severity [4] and portends a poor prognosis [9]. In other human studies, correlation network analysis identified a cilia gene module upregulated in IPF [35]. Several studies in mouse models also support a role for MMP7 in airway epithelial ciliogenesis. MMP7 restrains ciliated cell formation in the airway epithelium [28]. Ciliated cells carpet the airways after injury in MMP7 null mice [28].

MMP7 is constitutively expressed at low levels in peribronchial glandular cells and conducting airways in normal lung [17]. After injury, MMP7 expression is increased in lung epithelial cells; in human cystic fibrosis biopsy samples, MMP7 was noted to be expressed in alveolar type II cells as well as cells of the upper airway [17]. As the *Atp8b1* mouse exhibits higher expression of MMP7 with age (and without direct injury or insult to lung epithelia), it is possible that this mouse has innate epithelial repair defects. We focused on ciliated cells here, but it will be important to also examine alveolar areas for expression of MMP7.

As shown here, MMP7 is upregulated at both the transcript and protein levels in the *Atp8b1* mutant mouse, which develops pulmonary fibrosis, a phenotype accelerated by hyperoxia [22]. These results, along with the apical defects observed in lung epithelia and patchy parenchymal fibrosis after hyperoxic insult, make the *Atp8b1* mutant mouse a valuable model for studying IPF. Future work will examine the effects of other injurious stimuli, such as bleomycin, to provide comparison with existing models.

Humans with the *Atp8b1* mutation have progressive intrahepatic cholestasis and are prone to pneumonias [27]. The mechanism behind lung disease in these subjects remains incompletely understood. Known substrates of *Atp8b1* include phosphatidylserine and phosphatidylcholine, components of the plasma membrane. *Atp8b1* has been localized to the apical aspect of the plasma membrane [36]. Apical defects in the plasma membrane have been demonstrated in multiple cell types in *Atp8b1*-deficient cells, including stereocilia [26] and hepatocytes [25]. Apical defects observed in bronchial epithelial cells in the Apt8b1 mouse model corroborate these findings. *Atp8b1* also plays a role in apical membrane localization of proteins including Cdc42 [37] and CFTR [38].

The reason for the apical defects in lung epithelia, biliary epithelia and stereocilia is not known, but is thought to involve mislocalization of Cdc42 (cell division control protein 42 homologue), a Rho-family GTPase primarily involved in cellular polarization. A previously published transcriptome analysis of the *Atp8b1* mouse identified RhoA signaling as a canonical pathway [29] and other studies support a role for Cdc42 in apical defects in *Atp8b1*-deficient cells [37]. *Atp8b1* is thought to tether the small Rho-family GTPase Cdc42 at the apical membrane via the flippase’s preferred substrate, phosphatidylserine [37]. Loss of *Atp8b1* is thought to result in mislocalization of Cdc42 and may also explain the irregular apical shape of club cells or the truncation of ciliated cells seen on electron microscopy, as Cdc42 is primarily involved in cell polarization. Rho-family GTPases are essential in cellular motility and regulation of the actin cytoskeleton. Compelling evidence suggests that the *Atp8b1* flippase is required for Cdc42 clustering and cell polarization [39].

In summary, microarray and IPA data support decreased expression of ciliogenesis genes in the *Atp8b1* mutant mouse. The lung epithelial morphologic differences reported here also suggest a problem with ciliated cell differentiation or regeneration. These observations on TEM, along with parenchymal changes reported previously by our group, support the use of the *Atp8b1* mouse model for studying lung fibrosis [22]. Increased transcription and expression of MMP7 at the mRNA and protein level, respectively, in *Atp8b1* mouse vs. WT, warrants further exploration. The mechanism by which MMP7 may lead to loss of epithelial integrity and eventual lung fibrosis is currently under investigation, and the *Atp8b1* mouse model will provide the background on which to explore this mechanism.

Limitations of this study include the small number of animals (*n* = 3 *Atp8b1* mutant and *n* = 3 wild type), and the lack of a quantitative fibrosis score.

Pharmacologic inhibition of matrix metalloproteinases is notoriously difficult because of similarities in the catalytic region of various MMPs. Previous studies have shown side effects with broad MMP inhibition, specifically musculoskeletal side effects, possibly attributed to unintended metal chelation. In 2021, Mohan et al. reported a new anti-MMP7 antibody. More work is needed to understand the mechanistic role of MMP7 in IPF and the potential therapeutic targets thereof.

## Figures and Tables

**Table 1:** Differentially expressed genes associated with ciliogenesis in *Atp8b1* mutant mouse lung vs C57BL6 at 14 M (*q* < 0.1, mean–value difference between two groups >1.0).

Affymetrix Probe Set ID	Gene	Gene Name	Fold Change	*p* Value	q_MW (FDR)
1425291_at	*Foxj1*	Forkhead box J1	−1.205	0.026	0.0495
1450441_at	*Dnaaf1*	dynein, axonemal assembly factor 1	−1.02	0.026	0.0495
1455279_at	*Drc1*	dynein regulatory complex subunit 1	−1.288	0.0152	0.0357
1438466_at	*Dnah7b /// Dnah7c*	dynein, axonemal, heavy chain 7B /// dynein, axonemal, heavy chain 7C	−1.123	0.0152	0.0357
1438763_at	*Dnah2*	dynein, axonemal, heavy chain 2	−1.144	0.00866	0.026
1442894_at	*Dnah6*	dynein, axonemal, heavy chain 6	−1.322	0.026	0.0495
1453159_at	*Efhc1*	EF-hand domain (C-terminal) containing 1	−1.029	0.026	0.0495
1455379_at	*Dnali1*	dynein, axonemal, light intermediate polypeptide 1	−1.101	0.00433	0.0182

**Table 2:** Ingenuity Pathway Analysis; top canonical pathways identified in *Atp8b1* mutant lungs—transcripts associated with ciliogenesis.

Diseases or Functions Annotation	*p*-Value	Transcripts Associated with Ciliogenesis
Primary ciliary dyskinesia	3.56 × 10^−9^	*Dnaaf1, Dnah11, Dnal1, Drc1*
Movement of cilia	2.44 × 10^−7^	*Dnaaf1, Dnah11, Dnah7*
Abnormal morphology of cerebral ventricles	4.38 × 10^−5^	*Cetn2, Efhc1, Foxj1*
Situs inversus totalis	6.00 × 10^−5^	*Dnah11, Foxj1*
Patterning of left/right axis	1.62× 10^−4^	*Dnah11, Foxj1*
Formation of cilia	2.83× 10^−4^	*Cetn2, Dnaaf1, Foxj1*

## Data Availability

MIAME compliant microarray data was submitted to Gene expression omnibus (GEO) database and the assigned GEO Accession Number is (GSE80680) [29].
